# *In Vivo* Anti-Tumor Activity of Polypeptide HM-3 Modified by Different Polyethylene Glycols (PEG)

**DOI:** 10.3390/ijms12042650

**Published:** 2011-04-19

**Authors:** Zhendong Liu, Yinling Ren, Li Pan, Han-Mei Xu

**Affiliations:** Department of Marine Pharmacy, College of Life Science and Technology, China Pharmaceutical University, Nanjing 210009, China; E-Mails: liuzhendong0403@163.com (Z.L.); renyinling123@126.com (Y.R.); panli0225@163.com (L.P.)

**Keywords:** PEG modification, peptides, HM-3, anti-tumor, activities, ALD-mPEG (methoxy-polyethylene glycol propionaldehyde); molecular weight 10 kDa; named ALD-mPEG_10k_, SC-mPEG (α-methoxy-polyethylene glycol-ω-Succinimidyl Carbonate); molecular weight 20 kDa, named SC-mPEG_20k_

## Abstract

HM-3, designed by our laboratory, is a polypeptide composed of 18 amino acids. Pharmacodynamic studies *in vivo* and *in vitro* indicated that HM-3 could inhibit endothelial cell migration and angiogenesis, thereby inhibiting tumor growth. However, the half-life of HM-3 is short. In this study, we modified HM-3 with different polyethylene glycols (PEG) in order to reduce the plasma clearance rate, extend the half-life in the body, maintain a high concentration of HM-3 in the blood and increase the therapeutic efficiency. HM-3 was modified with four different types of PEG with different molecular weights (ALD-mPEG_5k_, ALD-mPEG_10k_, SC-mPEG_10k_ and SC-mPEG_20k_), resulting in four modified products (ALD-mPEG_5k_-HM-3, ALD-mPEG_10k_-HM-3, SC-mPEG_10k_-HM-3 and SC-mPEG_20k_-HM-3, respectively). Anti-tumor activity of these four modified HM-3 was determined in BALB/c mice with Taxol as a positive control and normal saline as a negative control. Tumor weight inhibition rates of mice treated with Taxol, HM-3, ALD-mPEG_5k_-HM-3, ALD-mPEG_10k_-HM-3, SC-mPEG_10k_-HM-3 and SC-mPEG_20k_-HM-3 were 44.50%, 43.92%, 37.95%, 31.64%, 20.27% and 50.23%, respectively. Tumor inhibition rates in the Taxol, HM-3 and SC-mPEG_20k_-HM-3 groups were significantly higher than that in the negative control group. The efficiency of tumor inhibition in the SC-mPEG_20k_-HM-3 group (drug treatment frequency: once per two days) was better than that in the HM-3 group (drug treatment frequency: twice per day). In addition, tumor inhibition rate in the SC-mPEG_20k_-HM-3 group was higher than that in the taxol group. We conclude that SC-mPEG_20k_-HM-3 had a low plasma clearance rate and long half-life, resulting in high anti-tumor therapeutic efficacy *in vivo*. Therefore, SC-mPEG_20k_-HM-3 could be potentially developed as new anti-tumor drugs.

## 1. Introduction

HM-3 is a new anti-tumor polypeptide developed in our laboratory, which is composed of 18 amino acids and previous studies showed that the target of HM-3 is integrin (αvβ3) [1]. *In vivo* and *in vitro* studies have shown that HM-3 could inhibit endothelial cell migration and angiogenesis, thereby inhibiting tumor growth. HM-3 is expected to be developed as therapeutic drugs for the treatment of solid tumors including gastric and liver cancer [2]. HM-3 has been authorized as a national invention patent and declared for an international patent [3].

However, similar to other peptide drugs, the half-life of HM-3 is short (approximately 25 min). HM-3 is easily degraded by proteases [1,4]. Animal studies suggested that the optimal therapeutic efficiency could be obtained by administration of the drug twice a day.

An effective approach to solve this problem is to modify peptide drugs with polymers such as polyethylene glycol (PEG) and dextran [5]. Currently, most studies are focusing on PEG modification [6–8]. PEG is a class of large polymers with unique physical and chemical properties. PEG is non-toxic, non-antigenic and has good biocompatibility [9,10]. The main biological function of PEG-modified protein or peptide drugs remains unchanged. More importantly, PEG modification increases the circulatory stability, extends the half-life, reduces the immunogenicity and decreases the toxcity of protein or peptide drugs [11–14].

However, PEG modification still exhibits several problems in application. Based on the literature [9,10], the main problems associated with PEG modification were an abnormal increase of molecular weight of the modified products and a decrease or loss of activity during the process of modification. Loss of activity is essentially the primary problem that needs to be solved for PEG modification of peptide drugs [15,16].

Selection of the appropriate molecular size of PEG is key for the modification of peptide drugs. Two main considerations for selecting the type of PEG are the residual *in vivo* biological activity and the renal elimination rate of the modified products. The molecular size of PEG is negatively related to the residual biological activity of the peptide drugs [17]. The relationship between the size of PEG and renal elimination rate is that a larger size of PEG normally results in a lower renal elimination rate. Studies have shown that the renal elimination rate when using PEG with a molecular weight of 20 kDa is approximately 10%, which plays a significant role in increasing the half-life, extending drug administration frequency and maintaining the blood drug concentration at a high level [18,19]. Currently, a variety of PEG-modified protein and peptide drugs have been developed and approved by the FDA for biomedical purposes [20].

ALD-mPEG and SC-mPEG are two different types of PEG, which are currently the most commonly used and relatively mature with active groups to modify polypeptide drugs. The active groups of ALD-mPEG and SC-mPEG are propionaldehyde and succinimidyl carbonate, respectively, which are specificity reactive groups of the peptide’s end amino. Physical properties, reaction activity with the peptide’s end amino of ALD-mPEG and SC-mPEG were different, and the remaining bioactivity of the same peptide PEGylated by the different two PEG were different too. A previous study [1] reported that HM-3 modified with ALD-mPEG_10k_ showed improved half-life and biological activity *in vitro*, but the half-life improvement was not significant.

In order to solve the problem of short half-life and rapid plasma clearance, our laboratory utilized four different types of PEG (ALD-mPEG and SC-mPEG) with different molecular weights to modify the *N*-terminus of HM-3. We expected that the modified products would have a long half-life while maintaining the anti-tumor activity. Our results showed that the modified product, SC-mPEG_20k_-HM-3, had a low plasma clearance rate, long half-life and retained its anti-tumor activity excellently. These results provide a fundamental basis for the development of new anti-tumor drugs.

## 2. Materials and Methods

### 2.1. Materials

#### 2.1.1. Cell Line and Animals

Human hepatic carcinoma SMMC-7721 cell line was purchased from American type Cell Culture (ATCC, Shanghai, China) and maintained in Dulbecco’s modified Eagle’s medium (DMEM) with 10% fetal bovine serum and antibiotics. BALB/c nude female mouse (SPF grade, 4–5 w, 20.9 ± 0.492 g) were purchased from the Shanghai Laboratory Animal Center of the Chinese Academy of Sciences.

#### 2.1.2. Main Reagents and Drugs

ALD-mPEG_5k_ (98% purity), ALD-mPEG_10k_ (100% purity), SC-mPEG_10k_ (98% purity), and SC-mPEG_20k_ (100% purity) were purchased from Beijing Kaizheng Biotech Development Co. Ltd. Batch No. were KZ-M05ALD-091207, KZ-M10ALD-091208, KZ-M10SC-091205, and KZ-M20SC-091206, respectively.

Trifluoroacetate (TFA), acetonitrile (ACN), and sodium cyanoborohydride were purchased from TEDIA, ROE Scientific Inc., and Shanghai Darui Fine Chemicals Co. Ltd, respectively.

HM-3 was chemically synthesized by GL Biochem (Shanghai), Ltd. The purity of the products was more than 99% by analytical high-performance liquid chromatography (HPLC), Batch No. 091020.

Mouse anti-HM-3 monoclonal antibody was prepared in our laboratory (density: 1.19 mg/mL, tite: 2,048,000). Horseradish peroxidase-labeled goat anti-mouse secondary antibody was purchased from Boster Biological Company of China.

Docetaxel was purchased from Hengrui Medicine Co. Ltd, Jiangsu province of China, Batch No. 10012812.

### 2.2. Methods

#### 2.2.1. PEG Modification of HM-3 and Purification

The forms of PEG modified HM-3 and the optimal conditions have been systematically studied [1,4,21]. As to the modification of HM-3 with ALD-mPEG_5k_ and ALD-mPEG_10k_, the optimal reaction time was over night at 4 °C; whereas the optimal time for SC-mPEG_10k_ and SC-mPEG_20k_ was 3 h at 4 °C. After the modification experiments, the products were purified by a semipreparative RP-HPLC and the purity was detected with analytical RP-HPLC. The reaction buffers and molar ratios (PEG:HM-3) are shown in Table 1.

#### 2.2.2. SDS-PAGE Electrophoresis and Western Blot Analysis of the Modified Products

Reaction mixtures were separated on a SDS-PAGE gel which was comprised of a 5% stacking gel and a 10% separation gel. The lane of the marker was stained with Coomassie brilliant blue and the lanes for ALD-mPEG_5k_-HM-3, ALD-mPEG_10k_-HM-3, SC-mPEG_10k_-HM-3 and SC-mPEG_20k_-HM-3 were stained with BaI_2_.

After SDS-PAGE electrophoresis, ALD-mPEG_5k_-HM-3, ALD-mPEG_10k_-HM-3, SC-mPEG_10k_-HM-3 and SC-mPEG_20k_-HM-3 were further identified by Western blot analysis using nitrocellulose membranes. The immunological reaction of ALD-mPEG_5k_-HM-3, ALD-mPEG_10k_-HM-3, SC-mPEG_10k_-HM-3 and SC-mPEG_20k_-HM-3 were performed on sections using a specific mouse anti-HM-3 monoclonal antibody of high titer and specificity and subsequently with horseradish peroxidase-labeled goat anti-mouse secondary antibody. Immunodetection was visualized using enhanced chemiluminescence.

#### 2.2.3. Inhibitory Effect of the Modified Products on the Growth of Human Hepatoma Cells *in Vivo*

SMMC-7721 cells were cultured in DMEM medium under 5% CO_2_/saturated humidified air at 37 °C. Cells were digested with the mixture of the trypsin (0.25%) and EDTA (0.02%) at logarithmic growth phase and passaged every 3–4 day. Then SMMC-7721 cells at logarithmic growth phase were digested to make a cell suspension (1.0 × 10^7^/mL), and 0.1 mL was injected into axilla of the left arm of BALB/c nude mouse. When tumor volume grew to 50 mm^3^–60 mm^3^, the mice were divided into 7 groups with 6 in each group. The doses and frequencies of drug administration are presented in Table 2 for the groups of paclitaxel, HM-3, ALD-mPEG_5k_-HM-3, ALD-mPEG_10k_-HM-3, SC-mPEG_10k_-HM-3 and SC-mPEG_20k_-HM-3.

All the mice (20 g) were administrated intravenously for 21 days and the tumor volumes were measured every two days. The solid tumor tissues were taken out the next day after the administration menu and the tissues were weighed to determine the inhibitory effects of the modified products. The calculation formula of tumor volume (*TV*) was as follows [22]: *TV* = 0.52 × *a* × *b*^2^, *a* and *b* indicate tumor length and width, respectively. Antitumor ratio determined by the tumor volumes is (*V* − *V_t_*)/*V* (*V* is the average tumor volume of the control group, and *V_t_* is the average tumor volume of the treated group). The antitumor ratio by the tumor weights was also calculated as (*W* − *W_t_*)/*W* (*W* is the average tumor weight of the control group, and *W_t_* is the average tumor weight of the treatment group).

#### 2.2.4. Statistical Methods

The data was analyzed using the statistics software SPSS13.0, which are expressed as mean ± SD. Statistical significance was assessed using the Student *t test*. For all statistical comparisons, treated groups were compared with normal sodium treated controls, and *P* < 0.05 was considered statistically significant; *P* < 0.01 was considered statistically very significant.

## 3. Results

### 3.1. Purity of the Four Modified HM-3 Products

**Figure 1.**
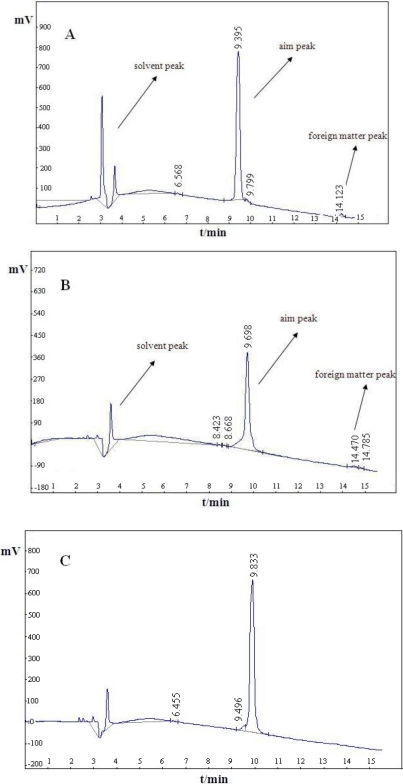

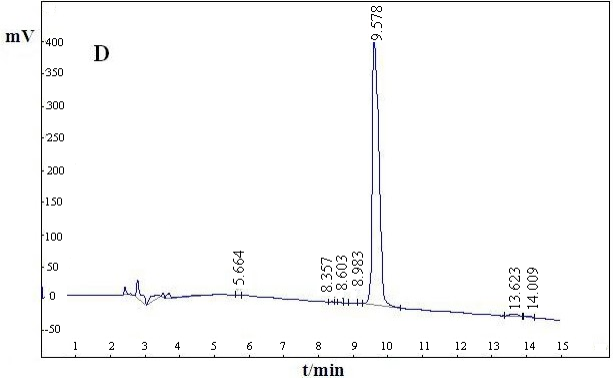
RP-HPLC analysis of PEGylated HM-3. Analysis was performed on a C18 column with water/ACN/TFA as eluent and a flow rate of 1 mL/min, and peaks were monitored at 220 nm. (**A**) ALD-mPEG_5k_-HM-3 purity: 97.39%, T: 9.395 min; (**B**) ALD-mPEG_10k_-HM-3 purity: 98.23%, T: 9.698 min; (**C**) SC-mPEG_10k_-HM-3 purity: 97.02%, T: 9.883 min; (**D**) SC-mPEG_20k_-HM-3 purity: 98.52%, T: 9.578 min.

RP-HPLC analysis showed that the purity of the four PEG-modified HM-3 products (ALD-mPEG_5k_-HM-3, ALD-mPEG_10k_-HM-3, SC-mPEG_10k_-HM-3 and SC-mPEG_20k_-HM-3) was over 97% (Figure 1).

### 3.2. SDS-PAGE and Western Blot Analysis of the Modified HM-3 Products

**Figure 2.**
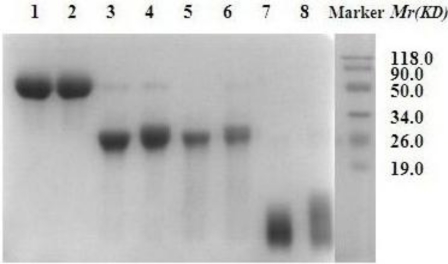
SDS-PAGE analysis of PEGylated HM-3. Lane Marker, Molecular weight markers; Lane 1, SDS-PAGE of SC-mPEG_20k_; Lane 2, SDS-PAGE of SC-mPEG_20k_-HM-3; Lane 3, SDS-PAGE of SC-mPEG_10k_; Lane 4, SDS-PAGE of SC-mPEG_10k_-HM-3; Lane 5, SDS-PAGE of ALD-mPEG_10k_; Lane 6, SDS-PAGE of ALD-mPEG_10k_-HM-3; Lane 7, SDS-PAGE of ALD-mPEG_5k_; Lane 8, SDS-PAGE of ALD-mPEG_5k_-HM-3.

**Figure 3.**
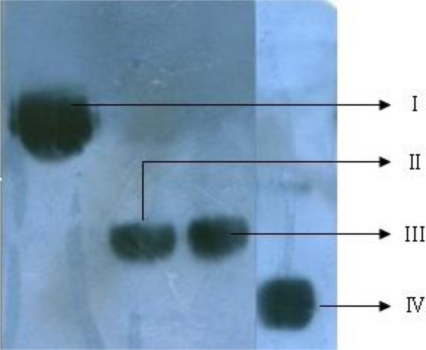
Western Blot analysis of PEGylated HM-3. (**I**) SC-mPEG_20k_-HM-3; (**II**) SC-mPEG_10k_-HM-3; (**III**) ALD-mPEG_10k_-HM-3; (**IV**) ALD-mPEG_5k_-HM-3.

Modification of HM-3 with mPEG resulted in mPEG-HM-3 with relatively high purity. SDS-PAGE analysis showed that the *Mr* of the modified product mPEG-HM-3 was much higher than that of the sum of HM-3 and mPEG (Figure 2). This could be due to the following two reasons. Firstly, linear PEG could extend on the surface of the protein, reducing the protein migration rate. Secondly, PEG had a very high degree of hydration, which increased its molecular size and reduced the migration rate in SDS-PAGE. We also found that monoclonal antibody binding site of the modified HM-3 were not covered by mPEG, which may be favorable for the maintenance of anti-tumor activity (Figure 3).

### 3.3. Anti-Tumor Activity of the Different PEG-Modified HM-3

**Figure 4.**
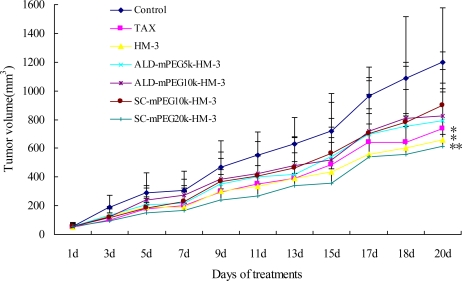
Therapeutic effects of peptides on the growth of tumors in BALB/c nude mice. Each point represents mean ±SD of each group (mean ± SD, n = 6, *P < 0.05; **P < 0.01 *versus* control). Control, once a day; The positive control drug Taxol at the dose of 10 mg/kg twice a week; HM-3 at the dose of 3 mg/kg twice a day; ALD-mPEG_5k_-HM-3 at the dose of 11.4 mg/kg once a day; ALD-mPEG_10k_-HM-3 at the dose of 19.9 mg/kg once every 2 day; SC-mPEG_10k_-HM-3 at the dose of 19.9 mg/kg once every 2 days; SC-mPEG_20k_-HM-3 at the dose of 36.7 mg/kg once every 2 day.

We treated nude mouse with HM-3, ALD-mPEG_5k_-HM-3, ALD-mPEG_10k_-HM-3, SC-mPEG_10k_-HM-3 and SC-mPEG_20k_-HM-3 and determined the tumor growth. Taxol was used as a positive control. We found that tumor growth in the HM-3 and SC-mPEG_20k_-HM-3 groups was significantly slower than that in the Taxol group. In addition, tumor growth in the HM-3 group and SC-mPEG_20k_-HM-3 group was significantly different from that in the negative control group (*P* < 0.05 and *P* < 0.01, respectively) (Figure 4).

**Figure 5.**
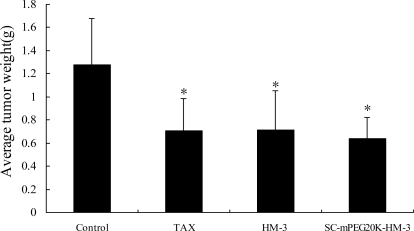
Average tumor weight in BALB/c nude mice. The data was analyzed using the statistics software SPSS13.0. Columns represent mean ± SD of each group (mean ± SD, *n* = 6, *P < 0.05 *versus* control).

**Figure 6.**
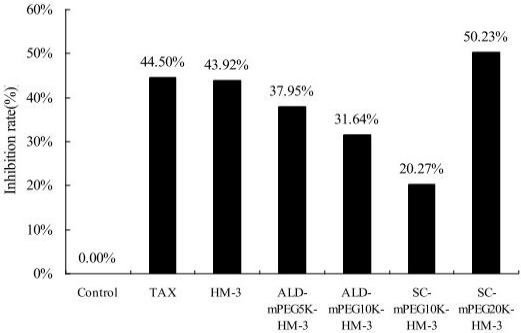
Inhibition rate of average tumor weight in BALB/c nude mice (*n* = 6).

**Figure 7.**
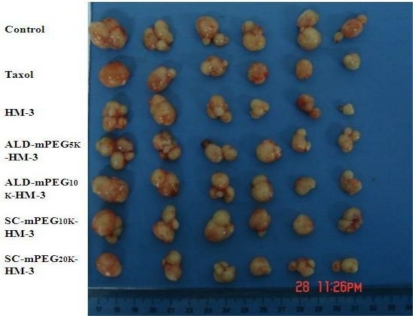
Photos of tumors at the end of the study indicating the therapeutic effects of peptides in BALB/c nude mice (*n* = 6).

**Figure 8.**
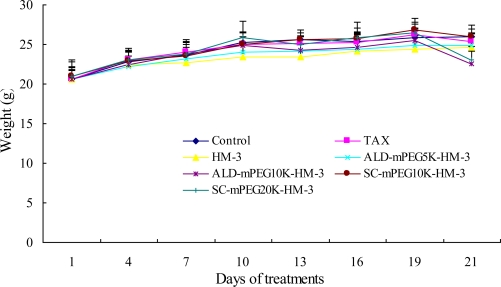
Weight change of BALB/c nude mice. Results are expressed as mean ±SD per group (mean ± SD, *n* = 6).

Tumor inhibitory rate in the SC-mPEG_20k_-HM-3 group (50.23%) and HM-3 group (43.92%) were similar to that in the Taxol group (44.50%), indicating that SC-mPEG_20k_-HM-3 maintained its anti-tumor activity. Tumor inhibitory rate in the ALD-mPEG_5k_-HM-3 and ALD-mPEG_10k_-HM-3 groups was 37.95% and 31.64%, respectively. Tumor weight in SC-mPEG_20k_-HM-3, HM-3 or Taxol groups was significantly different from that in the negative group (*P* < 0.05) (Figures 5 and 6). Photographs of the tumors at the end of the study are shown in Figure 7, tumors in the SC-mPEG_20k_-HM-3, HM-3 or Taxol groups were significantly different from that in the negative group. The body weight of nude mice in each group was stable (20–25 g), and was similar between different groups. Rapid increase or decrease of body weight did not occur (Figure 8).

## 4. Discussion

It is well known that attachment of polyethylene glycol (PEG) to therapeutic peptides or proteins (referred to as PEGylation) effectively prolongs their half-lives, reduces antigenicity and immunogenicity, and improves their pharmacokinetic and pharmacodynamic properties, rendering the drugs significantly prolonged circulating half-lives [23,24].

SC-mPEG (mPEG-succinimidyl carbonate) and ALD-mPEG (mPEG-propionaldehyde) have been the most commonly used chain-shaped PEG with active groups. Urethane bonds generated after coupling between SC-mPEG and peptides, and secondary amine generated after combining ALD-mPEG and peptides, are more stable and have a higher hydrolytic stability than ester bonds [25,26]. The SC-mPEG was extremely reactive, with reactions with peptides or proteins requiring higher pH in PBS, while the ALD-mPEG required mild reaction conditions, and could combine with peptides or proteins in mild pH in PBS [27]. Yet, the reactivity of the latter was lower than the former using the same peptides or proteins.

In PEGylation of pharmaceuticals, the design of an appropriate conjugation strategy is essential to confer the desired properties to the parent peptide or protein. The random conjugation of PEG would result in significant decrease, even loss of bioactivities, which showed inconsistent therapeutic effects and was undesired in PEGylated pharmaceuticals [20,28,29].

Therefore, when we modify a molecule with PEG, we should choose PEGs that have optimal balance between the activity maintaining rate and their half-life *in vivo*. According to previous studies, the *in vitro* activity of the PEGylated HM-3 products (especially SC-mPEG_20k_-HM-3) were high, but the *in vivo* activity was not measured. Thus, it is necessary to study the *in vivo* activity of the PEGylated HM-3 [1,4].

Figure 6 shows that the activity maintaining rate of modification by SC-mPEG_10k_ and ALD-mPEG_10k_ were not high, and the tumor inhibition rate of SC-mPEG_10k_-HM-3 (20.27%) was lower than ALD-mPEG_10k_-HM-3 (31.64%), while the modified product of SC-mPEG_20k_-HM-3 (50.23%) was excellent, displaying similar tumor inhibitory activity *in vivo* to the Taxol and unmodified HM-3 groups.

Generally speaking, modification with large molecule PEG may result in reduced activity. The activity of modified products of anti-tumor peptides and proteins is closely associated with organism resistance, PEG mass and shape, stability of PEG-peptide or PEG-protein bond, and changes in the spatial structure of modified products [27,30].

In our study, the activity of SC-mPEG_20k_-HM-3 did not change. This might be due to the fact that the loss of biological activity was compensated for by the significantly prolonged half-life and blood plasma residence time *in vivo*, as a result of the increased stability and higher hydrodynamic volume after modification. The activity of ALD-mPEG_5k_-HM-3, ALD-mPEG_10k_-HM-3, and SC-mPEG_10k_-HM-3 would decline with different degrees after modification, while the half-life and blood plasma residence time of these *in vivo* were low, which were not obviously prolonged in the previous study. Perhaps these factors cause the anti-tumor activity of ALD-mPEG_5k_-HM-3, ALD-mPEG_10k_-HM-3, and SC-mPEG_10k_-HM-3 to be lower than SC-mPEG_20k_-HM-3 and unmodified HM-3 *in vivo*. Besides, the active groups of SC-mPEG and ALD-mPEG are different, coupling between SC-mPEG and peptides with urethane bond, and in the presence of sodium cyanoborohydride, a secondary amine would be generated after combination of ALD-mPEG and peptides. The latter stability was higher than the former *in vivo* [31,32]. Maybe this led to the different activity of SC-mPEG_10k_-HM-3 and ALD-mPEG_10k_-HM-3 *in vivo*.

SC-mPEG_20k_-HM-3 retained the original biological activity of HM-3. Tumor inhibition of SC-mPEG_20k_-HM-3 (50.23%) was slightly higher than that of Taxol *in vivo*. These data suggest that the half-life of HM-3 can be improved by modification with SC-mPEG_20k_. For instance, drug administration frequency was extended from twice per day to once per two days after modification by SC-mPEG_20k_. The anti-tumor therapeutic efficacy of SC-mPEG_20k_-HM-3 was more efficient than that of HM-3. The body weight of mice in each group was maintained at a stable level, indicating that the effective dosage of drugs is non-toxic or the toxicity is low.

Therefore, this study demonstrated that SC-mPEG_20k_ can be used for modification of HM-3 and further determined *in vivo* biological activity of the modified product SC-mPEG_20k_-HM-3. We will further characterize the physio-chemical properties, *in vivo* pharmacokinetics, immunogenicity and the antitumor target of SC-mPEG_20k_-HM-3.

Our preliminary results found that although the tumor grew slowly and the tumor formation rate was low in nude mice inoculated with 5 × 10^5^ tumor cells/mice, administration of drugs had a good effect in inhibiting the tumors. The tumor inhibition for small tumors (20 mm^3^–30 mm^3^) was more efficient than for large tumors (>80 mm^3^). Taxol administered once per two days (10 mg/kg/time) had better tumor inhibition than that administered twice per week. However, Taxol is highly toxic and frequent administration of taxol may result in weight loss or even death. Thus, in this study, we treated the mice with Taxol twice per week. In addition, if the tumor cell line was transferred in culture too many times, the property of the cells may be changed, which may affect the evaluation of the therapeutic efficiency. These results provide references for other related studies.

## 5. Conclusion

SC-mPEG_20k_-HM-3 retained the anti-tumor activity of HM-3 better than other PEG-modified products. In addition, SC-mPEG_20k_ modified peptide HM-3 had a low plasma clearance rate, long half-life and retained its anti-tumor activity. Therefore, SC-mPEG_20k_-HM-3 has a very high value for development of new anti-tumor drugs.

## Figures and Tables

**Table 1.** HM-3, conditions of polyethylene glycol (PEG) modification.

**Phosphate Buffers, PBS (pH)**	**PEG**	**mol Ratio (PEG:HM-3)**	**Modification Rate**
PBS 5.0	ALD-mPEG_5k_	2:1	69.61%
PBS 5.0	ALD-mPEG_10k_	2.5:1	88.41%
PBS 8.0	SC-mPEG_10k_	1.5:1	95.94%
PBS 8.5	SC-mPEG_20k_	1.5:1	96.84%

**Table 2.** Dosage and frequency of drugs.

**Groups**	**Drugs**	**Frequency**	**Dosage**
First	Control	once a day	0.2 mL/d
Second	Taxol	twice a week	10 mg/kg
Third	HM-3	twice a day	3 mg/(kg·d)
Fourth	ALD-mPEG_5k_-HM-3	once a day	11.4 mg/(kg·d)
Fifth	ALD-mPEG_10k_-HM-3	every two days	19.9 mg/(kg·2d)
Sixth	SC-mPEG_10k_-HM-3	every two days	19.9 mg/(kg·2d)
Seventh	SC-mPEG_20k_-HM-3	every two days	36.7 mg/(kg·2d)

Annotation: PEG-HM-3 dosage is total PEG conjugate.
